# Restoration of Inferior Maxilla after Removal

**Published:** 1878-07

**Authors:** J. B. Hodgkin

**Affiliations:** Baltimore College of Dental Surgery.


### ARTICLE V.

### *Restoration of the Inferior Maxilla after Removal.*

[Reported by Prof. J. B. Hodgkin, Baltimore College of Dental Surgery.]

Dr. George B. Reynolds, Visiting Physician to "Bay View Asylum," and formerly Resident Physician of the Washington University Hospital, presented to the Class of the Baltimore College of Dental Surgery, Willie ——, aged eleven years, the child of German parents, for exhibition and remarks.

Dr. Reynolds said : The case before you, young gentlemen, is one of great interest, not only from its history, but from its rare occurrence. It is a case of necrosis of the lower maxilla, its successful removal, and the subsequent reproduction of that bone.

I need not explain to you the nature of necrosis of bone, and will only allude to some of the causes of the death of this bone under discussion. They may be from accident, as from blows upon the face, fractures, &c. ; the malady known as the phosphorus disease, to which those who work in match factories are most liable ; extensive mercurial salivation also may cause necrosis of this bone. It seems, sometimes, also to arise from causes which we call idiopathic, or, so to speak, of itself, having its origin in some depravity of the constitution.

Now, whether the case before us was a case of necrosis arising from mercurial salivation, or whether it arose primarily from some vice of this boy's constitution, some taint of the blood, it is difficult to decide; and though there are reasons for suspecting the first-named cause as bringing on the disease with its formidable results, yet I should rather be inclined to regard the case as one in which there exists a tendency to trouble—a condition of affairs only requiring occasion to develop the latent evil.

The history of this case is somewhat obscure. When Willie ——— first fell into the hands of Dr. H. B. Trist, who was then Professor of Anatomy in the Washington University School of Medicine, and myself, he had been ill for some time. His whole face was much swelled, indicating great inflammation of the parts, and there were seen three or four fistulous openings about the angle of the jaw, upon the left side, and down upon the neck, much such sinuses as you would see in suppurating scrofulous cervical glands. The fœtor from these discharging openings, and from others within the mouth, was so offensive that those having charge of him had left him to himself as far as possible, and he was the sole occupant of his room. He was in a most deplorable condition of general health, emaciated and worn down—his whole system so run down as to render the recovery of his health a problem, even should he be so fortunate as to get rid of his local disorder—the necrosed maxilla. You will understand that with all these outwardly discharging ulcers, and a still larger number discharging within the mouth, the pus being unavoidably swallowed with his food, and the constantly inhaled fœtor, that his condition was as deplorable as could well be.

The previous history of the case was so obscure as not to throw much light on it. The first notice, his mother says, of anything wrong, was a twisting of his mouth to one side, a condition we all recognize as facial paralysis, a lesion of one of the seventh pair of nerves. But we have no knowledge as to whether some previous lesion of the bones of the face had brought this trouble on by reflex action, or whether this was really the initial point of the subsequent serious trouble. Soon after this, he is reported as having been sick, and a doctor gave him some "whitish-grey powders" (most probably calomel, as there was the peculiar mercurial fœtor in the breath,) and that afterwards he had some trouble with his teeth, and that a dentist had extracted one or more. Then the trouble came on, in the depths of which we found him, as above stated. Had this dentist the acuteness that should characterize your profession, and could we find him now, he might throw some light upon this history; no one knows who he is, nor is it probable that his observations went further than the teeth to which his attention was called.

Our diagnosis of Willie's case was, that there was necrosis of the lower jaw, and in extent it seemed to comprise almost the entire bone. An operation was clearly indicated. It was absolutely necessary that the dead bone should be removed, but it was manifestly impossible to do this in his present physical condition, with his health all broken down; for such an operation he had not sufficient strength. Accordingly, he was put upon iron (the muriated tincture,) quinine and cod-liver oil, and these were used so successfully, that in a short time we had the satisfaction of seeing his appetite returning, his strength improving, and the whole constitution being renovated.

A most interesting question now arose as to the method of getting rid of the diseased and dead bone. The ordinary method of procedure is to remove it from without, an operation of formidable magnitude, involving the severance of the facial artery, and making a ghastly wound, and leaving an equally ghastly scar for life. And the idea was suggested, "can we not remove this dead bone from within, and by piece-meal, instead of at one operation?" This latter plan was adopted, and so successfully, that, at intervals extending over nearly a year, pieces of the bone were removed, as the case seemed to require it; removing the sequestra as they separated, making; for this purpose, incisions in the gum overlying the jaw. All this took a long time. As I have said, we were, perhaps, a year in getting out these pieces which I show you. The bone required amputation at the right angle of the jaw, and a little dissecting out at the left articulation. You observe from these pieces (and there are a good many of them, from very small spiculæ to this large piece, which includes the left ramus and candyle) that all the parts are crowded and honeycombed by the pus in which they were bathed so long. The sloughing had pretty effectively separated the bone from the soft parts.

So much for the history of the operation, and I only add that the soft parts healed readily.

But when I come to speak, as I now do, of the wonderful after-results in this case, you will understand why I class it as one of exceeding rarity. You all see for yourselves that the bone has been reproduced—that the boy has now a new jaw. True, the bone is somewhat smaller, and the chin a trifle shorter than before, and, as a matter of course, there are no teeth, save the solitary second right lower molar, which was left with the old bone of that side; and as the teeth anterior to this were erupted at the time of this operation they were removed also, and you see some of them in the bone I show you. The articulation of the candyle is perfect in its socket—there is no stiffness, no anchylosis—in a word, the jaw, except as to diminished size and length, is perfectly reproduced.

If I had any comment to make upon this case, I would simply say that you have here an evidence of the wonderful reparative powers of nature, and that these reparative powers seem nowhere so strongly marked as in recovery from injuries of the facial bones; and we see further, that the best surgery is to get out of nature's way—keep from interference with her—help her simply by removing obstacles; and that we should not despair of the most hopeless cases.

My friend, and your Prof. Hodgkin will now talk to you on the part of the case that falls into his specialty, and discuss the question as to whether the boy can have artificial substitutes for the lost teeth. The boy, as you see, looks to be in good health, and is fat and rosy.—*Va. Med. Monthly.*

